# Preparation of polypropylene/Mg–Al layered double hydroxides nanocomposites through wet pan-milling: non-isothermal crystallization behaviour

**DOI:** 10.1098/rsos.171070

**Published:** 2018-01-03

**Authors:** Yilei Zheng, Yinghong Chen

**Affiliations:** State Key Laboratory of Polymer Materials Engineering (Sichuan University), Polymer Research Institute of Sichuan University, Chengdu 610065, People's Republic of China

**Keywords:** polypropylene, layered double hydroxides, nanocomposites, crystallization, nucleation activity, effective activation energy

## Abstract

Differential scanning calorimeter was used to extensively investigate the non-isothermal crystallization of polypropylene (PP)/layered double hydroxides (LDHs) nanocomposites prepared through wet solid-state shear milling. The corresponding crystallization kinetics was further investigated by using Ozawa, modified Avrami and combined Avrami–Ozawa method, respectively. The results showed that the Ozawa method could not well describe the crystallization kinetics of pure PP and its nanocomposites. Comparatively, the modified Avrami method as well as the combined Avrami–Ozawa method gives the satisfactory results. Under the effect of pan-milling, the produced LDH nano intercalated/exfoliated particles exhibit the inhibitive effect on the PP nucleation but more remarkable promotion effect on the spherulite growth, leading to enhancement in the overall crystallization rate. This is reflected in increase of the calculated fold surface free energy *σ*_e_ and also the supercooling degree Δ*T* required for crystallization nucleation. In addition, the polarized optical microscopy observation also verifies the higher spherulite growth rate of PP/LDHs nanocomposites than that of pure PP.

## 1. Introduction

For many years, so much work has been done on the modification of polymers [1–5], because this would endow the polymer with the enhanced mechanical properties and the required functionalities such as magnetic, dielectric and optical properties. The compounding of the functional fillers such as barium titanate, carbon nanotube, graphene and clay with the polymer is the strategy generally adopted. As an inorganic layered compound, clay is extensively incorporated into polymer to prepare polymer/clay nanocomposites, which frequently exhibit some unexpected physico-chemical properties including reduced gas permeability, improved solvent resistance, superior mechanical performance, and enhanced flame retardant property. However, the above enhanced properties are rarely exhibited in the conventionally filled polymer composites and polymer blends [6–8]. For many years, much work has been focused on cationic clays such as montmorillonite-type layered silicate compounds [9]. However, there is also increasing attention paid to layered double hydroxides (LDHs) in the meantime [10–13]. The LDHs compound is a type of anionic or hydrotalcite-like clay, which consists of the positively charged brucite-like hydroxide layers and the hydrated exchangeable anions in the interlayer gallery [14,15]. Because LDHs have many favourable performances such as good catalytic property, flame retardancy, adsorption capability and tuneable composition and structure, they are increasingly applied to prepare the multifunctional nanocomposites [8] with polymers including polypropylene (PP).

In the prepared nanocomposites, various polymers are used [16–22], including PP, ethyl oleate, poly(l-lactide), polyoxymethylene, polyurethane, acrylonitrile–butadiene–styrene, polydimethylsiloxane, polyvinylpyrrolidone, etc. Comparatively, as a semicrystalline polymer, isotactic polypropylene (i-PP) has been widely used in many fields due to its cost-effective characteristics, such as good mechanical performance, heat resistance, fabrication flexibility and low cost, and is selected as the matrix in this paper. However, it has high flammability and relatively poor impact resistance especially at low temperatures, which limits its versatile applications to some extent [23]. Traditionally, compounding PP with inorganic nanoparticles is an effective method to improve the mechanical and thermal properties of PP. It is well known that the crystalline characteristics, such as crystalline morphology, crystallinity, spherulite size and crystallization rate, can affect the final properties of polymer to a considerable degree. Recently, there have been many studies on polymer crystallization by addition of different nanoparticles or nucleating agents to various polymer matrices [24–26]. In these studies, calcium carbonate [27], silicon dioxide [28], halloysite nanotubes [29], clay [26] and silicon nitride [30] are found to have the potential to act as nucleating agents for PP chain, which contributes to great improvement in the mechanical and thermal properties of the prepared PP composites. Maiti *et al*. [31] investigated the influence of crystallization on structure and morphology of PP/clay nanocomposites and found that the clay particles act as a nucleating agent for the matrix, but the linear growth rate or overall crystallization rate is not influenced much. Chen *et al*. [28] reported that SiO_2_ particles exhibit different nucleating effects on i-PP and co-PP, i.e. high nucleation activity for i-PP, but low nucleation activity for co-PP.

The studies on non-isothermal crystallization behaviour are currently receiving much attention. This is because the processing of most polymers is usually done under the non-isothermal crystallization conditions [32]. Therefore, the investigation of non-isothermal crystallization kinetics is necessary for interpreting the structure–property relationship of PP/LDHs nanocomposites. Ardanuy *et al*. [33] investigated the non-isothermal crystallization behaviour of PP/LDHs and PP/MMT nanocomposite and found that both LDHs and MMT particles play an active role in promoting the heterogeneous nucleation of PP and increased the overall crystallization rate. Lonkar *et al*. [34,35] investigated the isothermal and non-isothermal crystallization behaviour of PP/LDHs nanocomposites and found that the nano LDH layers can accelerate the overall crystallization process of PP as nucleating agents. Marega *et al*. [36] investigated the structure, morphology, thermal behaviour, physical property and crystallization behaviour of PP/LDHs nanocomposites, showing that LDHs enhance the rate of crystallization and facilitate the chain folding of macromolecules.

To the best of our knowledge, there are few studies conducted on the non-isothermal crystallization behaviour of PP/LDHs nanocomposites and particularly the effect of LDHs on spherulite growth rates. In most cases, in order to realize the intercalation or exfoliation of LDHs in PP matrix, the compatibilizer such as PP-g-MAH often needs to be added to PP/LDHs system. However, the addition of PP-g-MAH influences the crystallization of PP filled with LDHs to a considerable degree due to its accelerating crystallization effect on PP as a nucleating agent [37,38]. In our previous work [39], PP/LDHs nanocomposites were prepared successfully through wet solid-state shear milling (S^3^M) method without using any compatibilizer and organic modifier, which are generally required in the conventional preparation method such as melt intercalation, solution intercalation, *in situ* polymerization, exfoliation–adsorption process and template synthesis. This is the novelty in our technology. Obviously, our wet pan-milling method is very different from the conventional intercalated methods, so the prepared nanocomposite may show different crystallization behaviour. Based on the above background, in this paper, we continue to investigate the influence of LDH nanoparticles on the PP crystallization in the absence of compatibilizer. The non-isothermal crystallization kinetics of PP/LDHs nanocomposites, as well as the nucleation activity of LDHs is investigated on the basis of differential scanning calorimeter (DSC) method [40]. In addition, the effective activation energy of non-isothermal crystallization is also considered as a function of the relative crystallinity by using a differential isoconversional approach. The isoconversional approach is used to estimate the Hoffman–Lauritzen parameters (U* and *K*_g_) according to the overall rates of non-isothermal crystallization. Finally, the crystalline structure, morphology and spherulite growth rate of nanocomposites are also investigated by using polarized optical microscopy (POM).

## 2. Experimental

### 2.1. Materials

The isotactic PP, T30s, with diameter of 3–4 mm was supplied by the Dushanzi Petrochemical Co. (Kelamayi, China). MgAl-layered double hydroxides (hydrotalcite) were purchased from Jiangxi Hongyuan Chemical Co., Ltd (China). Sodium dodecyl sulfate was obtained from Chemical Reagent Factory of Kelong (Chengdu, China).

### 2.2. Equipment

The self-designed pan-mill type equipment was shown in the previous papers [41–43], which has the excellent pulverization and dispersion effects and can exert quite strong shear forces and pressures on the polymeric materials because of the ingenious design inspired by the traditional Chinese stone mill.

### 2.3. Preparation of PP/LDHs nanocomposites

The PP/LDHs co-powders prepared earlier [39] as master batch were diluted with neat PP to prepare PP/LDHs samples containing 0.5, 1, 2 and 3 wt% LDHs, respectively, in a twin-screw extruder (TSSJ-25) with *L*/*D* = 33 and *D* = 25 mm. The extrudate was pelletized, dried and then injection-moulded into testing samples in an injection moulding machine (K-TEC 40, Terromatik Milacron Corporation, Germany). The prepared PP/LDHs samples are named as PP/LS*x*, where *x* represents the content of LDHs in nanocomposites. The reference sample neat PP was obtained by following the same procedure. All the samples used in crystallization kinetics analysis were from the injection-moulded samples.

### 2.4. Characterization of crystallization behaviour

Non-isothermal crystallization kinetic measurements were carried out on a TA instruments Q20 DSC. Samples were heated from ambient temperature to 200°C at a rate of 10°C min^−1^ under a nitrogen atmosphere and kept there for 5 min to eliminate the influence of thermal history. After that, the samples were cooled to 40°C at the specified cooling rate (5, 10, 15 and 20°C min^−1^).

The spherulite growth rate was determined using a Leica DM2500P polarizing microscope (Leica Microsystems Inc., Germany). A thin sample sandwiched between two glass slides was first placed on the THMS600 Linkam heating stage and then heated to 200°C at a rate of 40°C min^−1^. After being at 200°C for 5 min, the sample was rapidly cooled to the selected crystallization temperature (125°C and 130°C) for isothermal crystallization. The crystallization processes were online observed and the morphological features were recorded at a constant time interval using PL-A662 video camera.

## 3. Results and discussion

### 3.1. Non-isothermal crystallization behaviour

**Figure 1.**
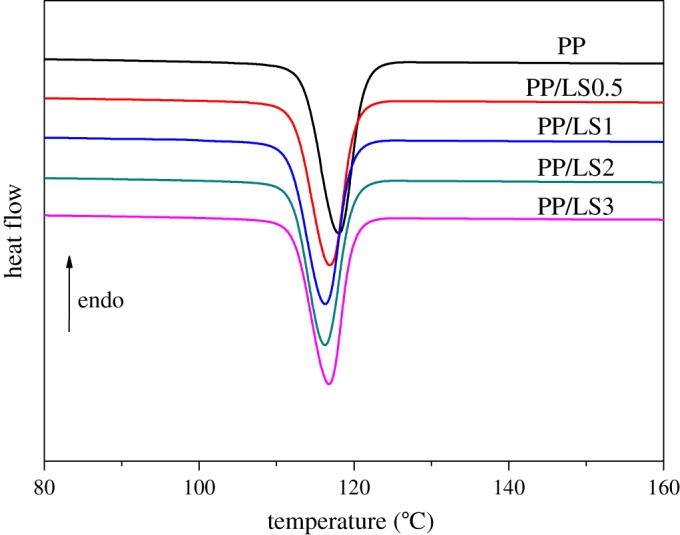
Crystallization DSC thermograms of pure PP, PP/LS0.5, PP/LS1, PP/LS2 and PP/LS3 samples at a cooling rate 10°C min^−1^. Reproduced with permission from Zheng & Chen [39] (Copyright © 2017 Royal Society of Chemistry).

In our previous work [39], we presented the non-isothermal crystallization results of different loading of LDHs incorporated PP nanocomposites prepared through wet solid-state shear milling method, as shown in figure 1. As a whole, relative to pure PP, the prepared PP/LDH nanocomposites have the lower crystallization peak temperature (*T*_p_). With increase of LDHs loading, the corresponding *T*_p_ continues to decrease but not obviously. However, at 3 wt% loading of LDHs, the *T*_p_ stops decreasing and increases slightly instead (still significantly lower than that of pure PP). The reason for this may be due to the reduction in the intercalation/exfoliation degree of LDHs at a relatively higher filler loading (3 wt%) to a certain degree. The above results show that incorporation of the intercalated/exfoliated LDH nano platelets formed under the effect of pan-milling prevents the nucleation of PP macromolecular chain and hence may postpone the crystallization to a certain degree, which is different from the literature reported [33–35], where the introduced LDHs are advantageous to the overall crystallization process of PP as the heterogeneous nucleating agent. However, the inhibitive effects of nanofillers on nucleation have also been reported in other polymer/inorganic fillers nanocomposites [24,27,44]. These are interesting results. In order to deeply investigate the difference between the crystallization behaviours of pure PP and PP/LDH nanocomposite samples with different LDH loading, PP, PP/LS0.5 and PP/LS3 samples were selected for DSC analysis at different cooling rate ranging from 5 to 20°C min^−1^.

**Figure 2.**
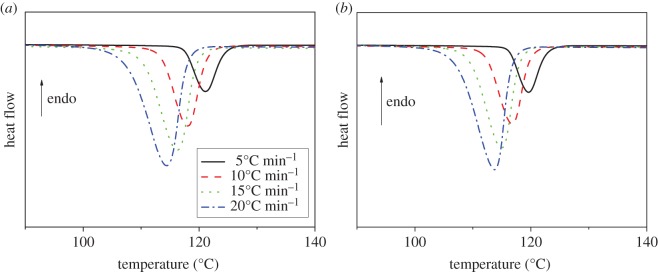
DSC thermograms of non-isothermal crystallization at various cooling rate: (*a*) pure PP and (*b*) PP/LS3.

Figure 2*a,b* shows the DSC crystallization curves of PP and PP/LS3 nanocomposites. The measured crystallization peak temperature (*T*_p_) and onset crystallization temperature (*T*_0_) at different cooling rates are shown in table 1. It can be seen that *T*_p_ would shift to a lower temperature and become broader with increasing cooling rates, indicating that the higher cooling rate would make PP macromolecular chains crystallize at the lower temperature. This means that the higher supercooling degree is required to initiate the crystallization at the faster cooling rate.

**Table 1.** The non-isothermal crystallization parameters *T*_p_, *T*_0_ and *t*_1/2_ of pure PP and PP/LDHs nanocomposites at various cooling rates (*a*).

sample	*α* (°C min^−1^)	*T*_p_ (°C)	*T*_0_ (°C)	*t*_1/2_ (min)
pure PP	5	121.1	127.54	1.38
	10	118.0	124.41	0.74
	15	116.2	122.59	0.53
	20	114.37	120.78	0.42
PP/LS0.5	5	120.02	126.34	1.36
	10	116.88	123.29	0.74
	15	115.44	121.53	0.52
	20	114.16	120.02	0.4
PP/LS3	5	119.61	125.61	1.28
	10	116.78	122.88	0.71
	15	114.91	121.00	0.5
	20	113.74	119.63	0.4

### 3.2. Kinetics of non-isothermal crystallization

The difference between the crystallization behaviours of pure PP and PP/LDH nanocomposites can be well illustrated by analysing the corresponding non-isothermal crystallization kinetics, which can be characterized by the dependence of relative crystallinity (*X*_T_) on crystallization temperature (*T*) and crystallization time (*t*), respectively.

**Figure 3.**
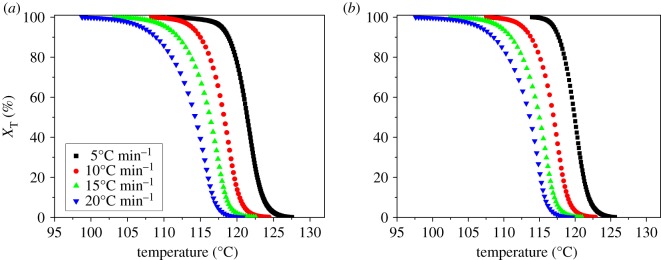
The relative crystallinity with temperature for the non-isothermal crystallization of (*a*) pure PP and (*b*) PP/LS3 at various cooling rates.

The relative crystallinity (*X*_T_) [45] as a function of crystallization temperature (*T*) can be expressed as follows:

3.1 $$X_{T}=\frac{\int_{T_{0}}^{T}(\text{d}H_{c}\text{/d}T)\text{d}T}{\int_{T_{0}}^{T_{\infty }}(\text{d}H_{c}\text{/d}T)\text{d}T},$$

where d*H*_c_ is the crystallization enthalpy released in an infinitesimal temperature range d*T*; *T*_0_, *T* and *T*_∞_ are the initial crystallization temperature, the crystallization temperature at time *t* and the final crystallization temperature, respectively. Figure 3 shows the *X*_T_ ∼ *T* relationship curves of pure PP and PP/LS3 sample at various cooling rates.

**Figure 4.**
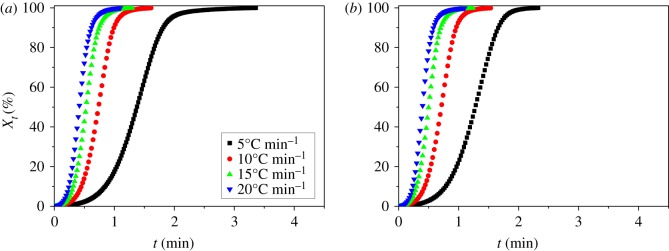
The relative crystallinity with time for the non-isothermal crystallization of (*a*) pure PP and (*b*) PP/LS3 at various cooling rates.

In addition, there is a relationship between crystallization time *t* and crystallization temperature *T*:

3.2 $$t=\frac{\left(T_{0}-T\right)}{\alpha },$$

where *α* is the cooling rate, *T*_0_ is the onset crystallization temperature and *T* is the temperature at crystallization time *t*. Combining equation (3.2) with equation (3.1), the corresponding *X*_T_ ∼ *t* relationship curves can also be obtained, as shown in figure 4.

The crystallization half-time (*t*_1/2_) (the time to reach 50% relative crystallinity) is also listed in table 1. It is apparent that *t*_1/2_ decreases with increasing cooling rate. However, at a given cooling rate, the crystallization half-time of PP/LDHs nanocomposites is slightly lower than that of PP and even further decreases with increasing the LDH content. These results indicate that the addition of LDH particles could facilitate the overall crystallization process, which seems to be inconsistent with the previous results [39] obtained, i.e. the addition of LDH would prevent the nucleation of PP macromolecular chain (may retard the crystallization). Obviously, this is not true. As we know, the overall crystallization rate is determined by both nucleation rate and spherulite growth rate. Hence, there is possibly more important positive factor in influencing the crystallization of PP/LDHs nanocomposite system in the non-isothermal crystallization process, which should be ascribed to the spherulite growth rate. In order to fully understand this point, we will deeply examine the corresponding crystallization mechanism of PP/LDHs system by further analysing the non-isothermal crystallization kinetics of pure PP and PP/LDHs sample based on Ozawa, modified Avrami and Avrami–Ozawa methods, respectively.

#### 3.2.1. Kinetics of non-isothermal crystallization by Ozawa method

According to Ozawa theory [46], the non-isothermal crystallization process can be regarded as a result of infinitesimally small isothermal crystallization steps. The relative crystallinity (*X*_T_) [46] can be accordingly written as a function of cooling rate *α*:

3.3 $$1-X_{T}=\exp\left(\frac{-K(T)}{\alpha^{m}}\right),$$

where *K*(*T*) is the cooling crystallization function that indicates how fast crystallization proceeds, and *m* is the Ozawa exponent depending on the dimension of crystal growth. According to equation (3.3), the double logarithmic form can be taken as follows:

3.4 $$\ln[-\ln(1-X_{T})]=\ln K(T)-m\;\ln\;\alpha .$$

**Figure 5.**
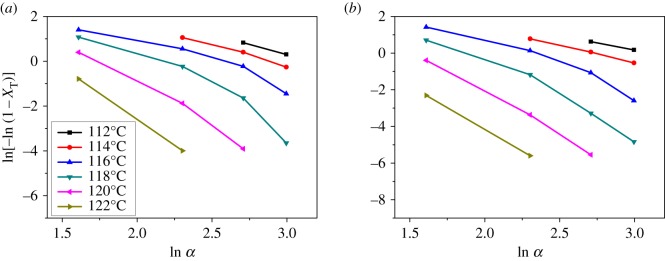
Ozawa plots of ln[−ln(1 − *X*_T_)] versus ln *α* for the non-isothermal crystallization of (*a*) pure PP and (*b*) PP/LS3.

By plotting ln[−ln(1 − *X*_T_)] versus ln *α* at a given temperature, a straight fitted line should be obtained if the Ozawa method is valid. Accordingly, the *K*(*T*) and *m* can be derived from the slope and the intercept, respectively. The fitted curves of PP and PP/LS3 are shown in figure 5. It can be seen that the plots of PP and its nanocomposite show deviation from linearity. This is probably due to the approximate assumptions in the Ozawa theory, e.g. ignoring the secondary crystallization, the dependence of the fold length on temperature and the constant value of cooling function over the entire crystallization process [47,48]. Therefore, the Ozawa method is not an effective way for describing the non-isothermal crystallization process of PP/LDHs nanocomposites.

#### 3.2.2. Kinetics of non-isothermal crystallization by modified Avrami method

The Avrami equation used to describe the non-isothermal crystallization kinetics can be expressed as follows [49,50]:

3.5 $$X_{t}=1-\exp(-Z_{t}t^{n}),$$

or

3.6 $$\ln[-\ln(1-X_{t})]=\ln\;Z_{t}+n\;\ln t,$$

where *X_t_* is the relative crystallinity at crystallization time *t*, *n* is the Avrami exponent associated with the crystallization mechanism and *Z_t_* is the crystallization rate constant related to both nucleation and growth rate parameters. Unlike isothermal crystallization, the parameters of *n* and *Z_t_* do not have the similar physical meaning for non-isothermal crystallization due to the change in temperature, but the direct application of the Avrami equation could still provide some insights into the description of the crystallization. Considering the non-isothermal crystallization process, Jeziorny [51] suggested that the rate parameter *Z_t_* should be corrected as follows:

3.7 $$\ln\;Z_{c}=\frac{\ln Z_{t}}{\alpha }.$$

**Figure 6.**
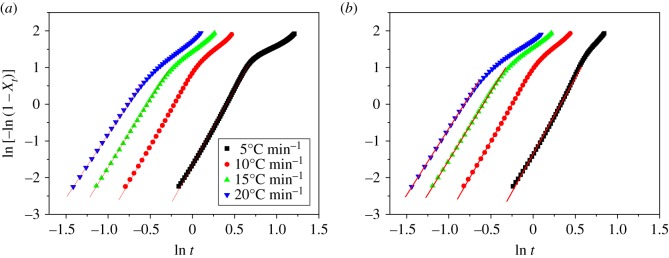
Avrami plots of ln[−ln(1 − *X_t_*)] versus ln *t* for the non-isothermal crystallization of (*a*) pure PP and (*b*) PP/LS3.

The values of *n* and *Z_t_* can be obtained from the slope and intercept of the plots of ln[−ln(1 − *X_t_*)] versus ln *t*, respectively, as shown in figure 6. It can be seen that straight lines could be obtained in each cooling rate at the early stage of crystallization. These results are listed in table 2 (figure 6 only gives the results of pure PP and PP/LS3 as examples; for clarity, the results of PP/LS0.5 are also included here). It can be seen that the calculated average values of Avrami exponent *n* are about 3.82, 3.92 and 3.87 for PP, PP/LS0.5 and PP/LS3, respectively. This indicates that LDHs do not exhibit nucleating effect and almost do not change the nucleation mechanism. The *Z*_c_ values of nanocomposites are slightly higher than that of pure PP at the same cooling rate, showing that incorporation of nano-LDHs could accelerate the overall crystallization process to a certain degree.

**Table 2.** The non-isothermal crystallization parameters by modified Avrami method.

sample	*α* (^o^C min^−1^)	*Z_t_*	*Z*_c_	*n*
pure PP	5	−1.66	0.72	4.12
	10	0.86	1.09	3.97
	15	1.98	1.14	3.71
	20	2.66	1.14	3.48
PP/LS0.5	5	−1.63	0.72	4.28
	10	0.84	1.09	3.99
	15	2.08	1.15	3.79
	20	2.94	1.16	3.62
PP/LS3	5	−1.35	0.76	4.14
	10	0.99	1.10	3.98
	15	2.21	1.16	3.74
	20	2.96	1.16	3.62

#### 3.2.3. Kinetics of non-isothermal crystallization by combined Avrami–Ozawa method

By combining the Ozawa and Avrami equations, a new method was proposed by Mo and co-workers [52] to describe the non-isothermal crystallization kinetics. Because the crystallinity is related to both the cooling rate *α* and the crystallization time *t* (or temperature *T*), the relation between *α* and *t* could be defined for a given crystallinity. Consequently, combining equations (3.4) and (3.6), a new kinetics model [52] is derived for non-isothermal crystallization:

3.8 $$\ln\;Z_{t}+n\;\ln t=K(T)-m\;\ln\alpha .$$

Equation (3.8) can be rewritten as follows:

3.9 ln⁡α=ln⁡F(T)−a ln⁡t,

where *F*(*T*) = [*K*(*T*)/*Z_t_*]^1/m^ and *a* = *n*/*m* (*n* is Avrami exponent and *m* is Ozawa exponent). Here, for non-isothermal crystallization, the physical meaning of *F*(*T*) is the necessary cooling rate value to reach a certain relative crystallinity of one polymer system in unit time. So, *F*(*T*) can be used as a parameter to measure the polymer crystallization rate.

**Figure 7.**
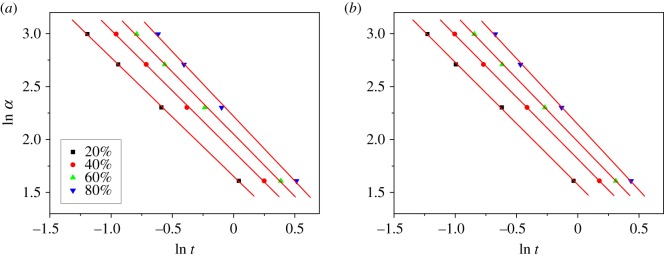
Combined Avrami–Ozawa plots of ln *α* versus ln *t* for the non-isothermal crystallization of (*a*) pure PP and (*b*) PP/LS3.

According to equation (3.9), at a given crystallinity, the plot of ln *α* versus ln *t* will yield a straight line with an intercept of ln *F*(*T*) and a slope of –*a*. Plots of ln *α* versus ln *t* at various crystallinity for PP and PP/LS3 are shown in figure 7. It is seen that a good linear relationship is obtained. This suggests that this analysis method is more effective in describing the non-isothermal crystallization kinetics of PP and its nanocomposite. The values of *F*(*T*) and *a* are listed in table 3 (figure 7 only gives the results of pure PP and PP/LS3 as examples; for clarity, the results of PP/LS0.5 are also included here). As can be seen, the value of *a* varies from 1.10 to 1.24 and the value of *F*(*T*) increases with increasing the relative crystallinity, indicating that in the unit crystallization time, a higher crystallinity obtained requires a higher cooling rate. In addition, the value of *F*(*T*) of PP/LDHs samples are lower than that of pure PP at a given crystallinity, indicating that the prepared PP/LDHs nanocomposites could crystallize faster than pure PP.

**Table 3.** Kinetic parameters at different relative crystallinity by combining Avrami–Ozawa method.

sample	*X_t_*(%)	*a*	*F*(*T*)
pure PP	20	1.12	5.22
	40	1.15	6.60
	60	1.18	7.77
	80	1.22	9.19
PP/LS0.5	20	1.10	5.23
	40	1.13	6.53
	60	1.16	7.64
	80	1.20	8.95
PP/LS3	20	1.15	4.83
	40	1.17	6.15
	60	1.20	7.24
	80	1.24	8.53

#### 3.2.4. Nucleation activity

Dobreva & Gutzow [53,54] suggested a simple method to calculate the nucleation activity *φ* of foreign substrates in polymer melt. Nucleation activity *φ* is a factor by which the work of three-dimensional nucleation decreases with the addition of a foreign substrate. The value of *φ* generally ranges from 0 to 1. The stronger the activity of the foreign substrates is, the lower the *φ* value could be. The nucleation activity *φ* is calculated according to the following equation [53]:

3.10 $$\phi =\frac{B^{*}}{B^{0}},$$

where B* stands for the parameter of heterogeneous nucleation, while *B*^0^ stands for the parameter of homogeneous nucleation. The involved *B* is a parameter that can be calculated according to the following equation [54]:

3.11 $$B=\frac{\omega \sigma^{3}V_{m}^{2}}{3kT_{m}\Delta S_{m}^{2}n},$$

where *k* is the Boltzmann constant, *σ* is the specific energy, *V*_m_ is the molar volume of one crystalline polymer, *S*_m_ is the entropy of melting, *T*_m_ is the PP melting temperature and *ω* is a geometrical factor.

Generally, the parameter *B* could be experimentally obtained from crystallization empirical correlation [54], i.e. the following relationship:

3.12 $$\log\alpha =\text{const}-\frac{B}{2.3\Delta T_{p}^{2}},$$

where *α* is the crystallization rate and Δ*T*_p_ is the supercooling degree (*T*_m_ − *T*_p_).

**Figure 8.**
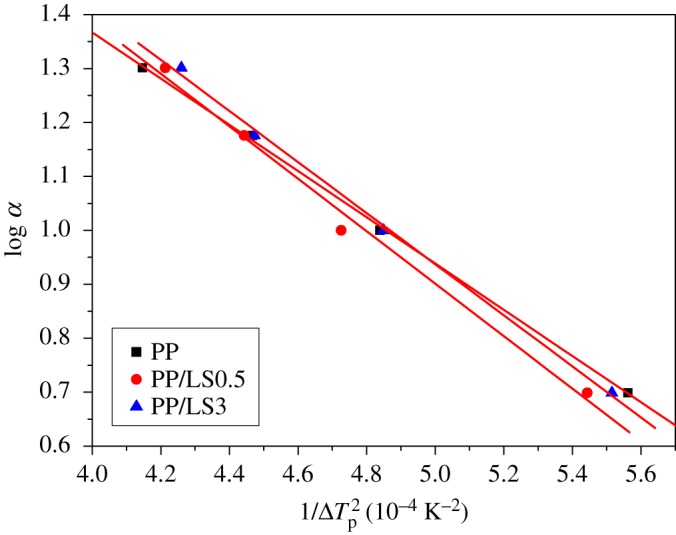
Plots of log *α* versus $1/\Delta {T_{p}}^{2}$ for pure PP, PP/LS0.5 and PP/LS3.

Plots of ln *α* versus $1/\Delta {T_{p}}^{2}$ for PP, PP/LS0.5 and PP/LS3 are shown in figure 8. As can be seen, some fitted straight lines are obtained. From the slope of these lines, the values of *B* are calculated. The calculated values of *φ* for PP/LS0.5 and PP/LS3 are 1.135 and 1.105, respectively, of which both are greater than 1. These results prove that the added LDHs particles assuredly have no heterogeneous nucleation effect and, further, would restrict the nucleation of PP macromolecular chains.

#### 3.2.5. Effective activation energy for non-isothermal crystallization process

For non-isothermal crystallization process, it is also important and interesting to evaluate effective activation energy, i.e. Δ*E*. Among the commonly used methods, the Kissinger method [55] has been one of the most popular approaches for evaluating the overall effective activation energy. However, Vyazovkin [56] demonstrated that this method which possibly gives the negative sign for *α* could produce mathematically invalid results that generally make the Kissinger equation inapplicable to the polymer melt crystallization process. To avoid the above problem, an isoconversional method can be generally applied to non-isothermal crystallization in evaluating the dependence of the effective activation energy on conversion and temperature [57,58]. Such dependencies have been quite helpful in detecting and elucidating complex kinetics in polymeric systems. There are three different isoconversional methods [59–61], i.e. the differential isoconversional method (e.g. Friedman method), the intergral isoconversional methods (e.g. Flynn and Wall, Ozawa method) and the advanced isoconversional method (e.g. Vyazovkin method). In these methods, the Friedman method was usually used due to its simplicity.

The Friedman equation [60] is expressed as follows:

3.13 $$\ln\;{\left(\frac{\text{d}X}{\text{d}t}\right)}_{X,i}=\text{const}-\frac{\Delta E_{X}}{RT_{X,i}},$$

where d*X*/d*t* is the instantaneous crystallization rate as a function of time at a given conversion *X*. According to this method, d*X*/d*t* can be obtained by using the differentiation of *X_t_* function derived from the integration of the experimentally measured crystallization rate with respect to time. In addition, by selecting appropriate crystallinity (e.g. from 2 to 98%), the d*X*/d*t* value at a certain *X* could be correlated to the corresponding crystallization temperature (*T*_x_). As a result, by plotting the left-hand side of equation (3.13) versus 1/*T*_x_, a fitted straight line should be obtained with a slope equal to Δ*E*_X_/*R*. Papageorgiou *et al*. [62] successfully used Friedman isoconversional method to estimate the dependence of effective activation energy on conversion of PP/surface-treated SiO_2_ system.

**Figure 9.**
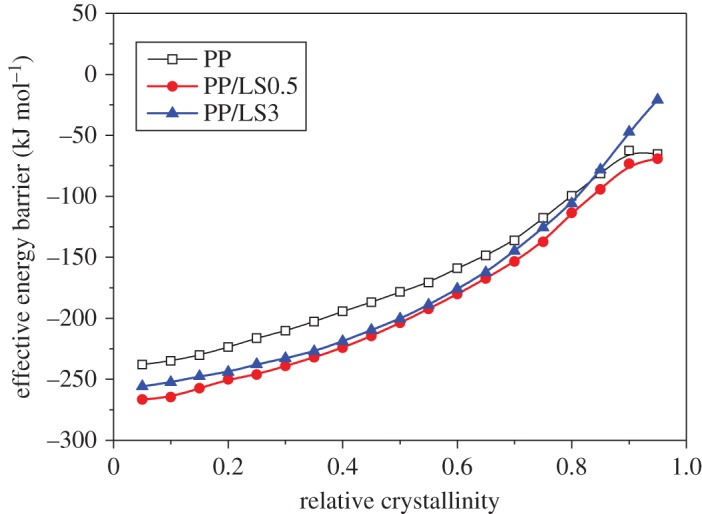
Dependence of the effective activation energy on the relative crystallinity using isoconversional analysis for PP, PP/LS0.5 and PP/LS3.

**Figure 10.**
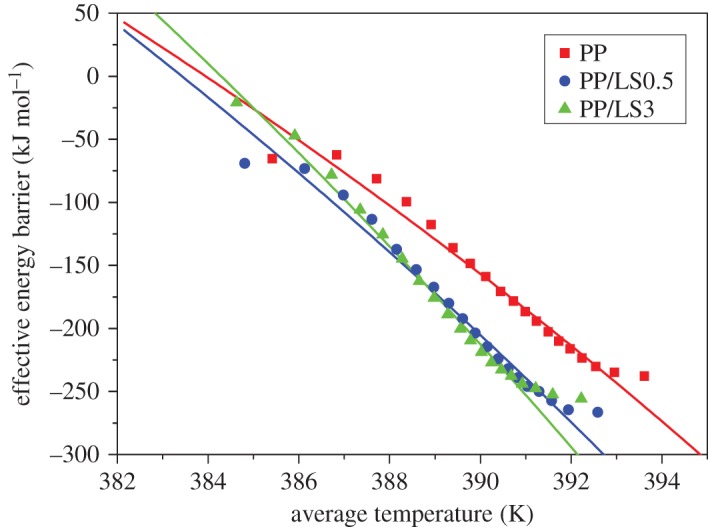
Dependence of the effective activation energy on the average temperature based on the isoconversional analysis for PP, PP/LS0.5 and PP/LS3.

The dependence of the effective activation energy on the relative crystallinity of PP and PP/LDHs nanocomposite is presented in figure 9. As can be seen, Δ*E* increases monotonically with the increase in the relative crystallinity. In addition, in all cases, Δ*E* shows the high absolute values at low conversion that correspond to the temperature closer to the melting point, indicating that the crystallization rate increases with decreasing temperature. This is the feature of the crystallization region that corresponds to the temperature higher than that of the maximum crystallinity. For a given conversion, it is observed that PP/LDHs nanocomposites show lower Δ*E* values than pure PP. These results imply that the presence of LDH particles improves the transportation capability of macromolecular chain segments to the growing surface of PP crystals, which means that PP chain segments require less energy to be rearranged in the presence of LDHs. As a result, the PP macromolecular chains in nanocomposite sample crystallize much faster. Furthermore, according to the studies in the literature [63,64], the effective activation energy can be plotted as a function of the average temperature associated with a certain relative crystallinity, as shown in figure 10. Such plots can be used in evaluating the Hoffman–Lauritzen parameters.

#### 3.2.6. Evaluation of Hoffman–Lauritzen parameters of non-isothermal crystallization

The crystallization thermodynamics and kinetics of polymer nanocomposites have been analysed on the basis of the Hoffman–Lauritzen secondary nucleation theory [65,66]. The crystal growth (*G*) [65] is given as a function of crystallization temperature (*T*), as follows:

3.14 $$G=G_{0}\;\exp\;\left[-\frac{U^{*}}{R(T-T_{\infty })}\right]\;\exp\;\left[-\frac{K_{g}}{T\Delta Tf}\right],$$

where *G*_0_ is the pre-exponential factor and U* is the activation energy of the chain segment movement. The first exponential term involves the contribution of the diffusion process and the crystal growth rate, while the second exponential term is contributed by the nucleation process; U* is the Vogel–Fulcher–Tamman–Hesse (VFTH) parameter describing the transport of polymer chain segments across the liquid/crystal interphase. In addition, *K*_g_ is a nucleation constant, Δ*T* = *T*_m_ − *T* is the supercooling degree, *f* = 2 *T*/(*T*_m_ − *T*) is the correction factor and *T*_∞_ is a hypothetical temperature where motion associated with viscous flow state almost ceases (usually taken as a value of 30 K below the glass transition temperature *T*_g_ [66]). In this study, the *T*_g_ value of pure PP is 270 K [62] and the equilibrium melting temperature *T*_m_ is calculated as 212.1°C using nonlinear Hoffman–Weeks extrapolation [67]. Furthermore, the kinetic parameter *K*_g_ [66] has the following form:

3.15 $$K_{g}=\frac{nb\sigma \sigma_{e}T_{m}}{\Delta h_{f}k_{B}},$$

where *n* takes the value 4 for crystallization regimes I and III and 2 for regime II [68], respectively (in this work, obviously, *n* = 4); *b* is the distance between two adjacent fold planes (for pure PP, *a* = 5.49 × 10^−10^ m, *b* = 6.26 × 10^−10^ m [67,68]), *σ* and *σ*_e_ are the lateral and fold surface free energy (*σ* = 0.1Δ*h*_f_(*ab*)^1/2^ = 11.31 erg/cm^2^), respectively, *T*_m_ is the equilibrium melting temperature, Δ*h*_f_ is the heat of fusion per unit volume of crystal (1.93 × 10^8^ J m^−3^) [69] and *k*_B_ is the Boltzmann constant (1.38 × 10^−23^ J K^−1^). By rearranging equation (3.14), the following correlation can be obtained:

3.16 $$\ln G+\frac{U^{*}}{R(T-T_{\infty })}=\ln\;G_{0}-\frac{K_{g}}{T\Delta Tf}.$$

The values of *K*_g_ can generally be determined by the linear plot of the left-hand side of equation (3.16) against (*T*Δ*Tf*)^−1^. The crystal growth rate *G* is often replaced by the reciprocal of time needed to reach a half of the relative crystallinity in isothermal crystallization process. According to Vyazovkin & Sbirrazzuoli [63], isoconversional analytical method was combined with the crystal growth rate theory of Hoffman–Lauritzen to propose a mathematical procedure which can allow the non-isothermal data on overall crystallization rates to be used for determining the parameters U* and *K*_g_. The classical Hoffman–Lauritzen equation was differentiated with respect to *T*^−1^ to derive a temperature dependence of the effective activation energy [63], as follows:

3.17 $$E_{X}(T)=-R{\left[\frac{\text{d}\;\ln(\text{d}X\text{/d}t)}{\text{d}T^{-1}}\right]}_{X}=-R\frac{\text{d}\;\ln G}{\text{d}T^{-1}}=U^{*}\frac{T^{2}}{{\left(T-T_{\infty }\right)}^{2}}+K_{g}R\frac{T_{m}^{2}-T^{2}-T_{m}T}{{\left(T_{m}-T\right)}^{2}T}.$$

Using this equation, the Hoffman–Lauritzen parameters can be calculated by performing the nonlinear fitting based on equation (3.17) by taking advantage of experimental data produced by Friedman differential isoconversional method. This method has been successfully used to estimate the Hoffman–Lauritzen parameters from the overall rates of the non-isothermal crystallization of poly(propylene terephtalate) and poly(butylene naphthalate) [70]. In the present work, the estimated values of U* and *K*_g_ are listed in table 4 and the values of *K*_g_ are then used for the determination of fold surface free energy. The results show that the fold surface free energy *σ*_e_ increases with the increasing LDHs content, which may be due to the decrease in the folding entropy resulting from the reduced mobility of the PP chains. This means that the formation of new nuclei needs more surface free energy in the presence of LDHs, i.e. showing the inhibitive nucleation effect. These results are consistent with the previous analyses. Therefore, the isoconversional method can be successfully employed for investigation of the non-isothermal crystallization behaviour of PP/LDHs nanocomposite system in this paper.

**Table 4.** The crystallization parameters of pure PP and its nanocomposites.

sample	U* (J mol^−1^)	*K*_g_ (10^5^ K^2^)	*σσ*_e_ (10^−3^ J^2^ m^−4^)	*σ*_e_ (erg cm^−2^)
pure PP	88 160	30.25	6.631	586.3
PP/LS0.5	105 200	36.83	8.074	713.9
PP/LS3	127 600	43.21	9.472	837.5

### 3.3. Spherulite growth behaviour

**Figure 11.**
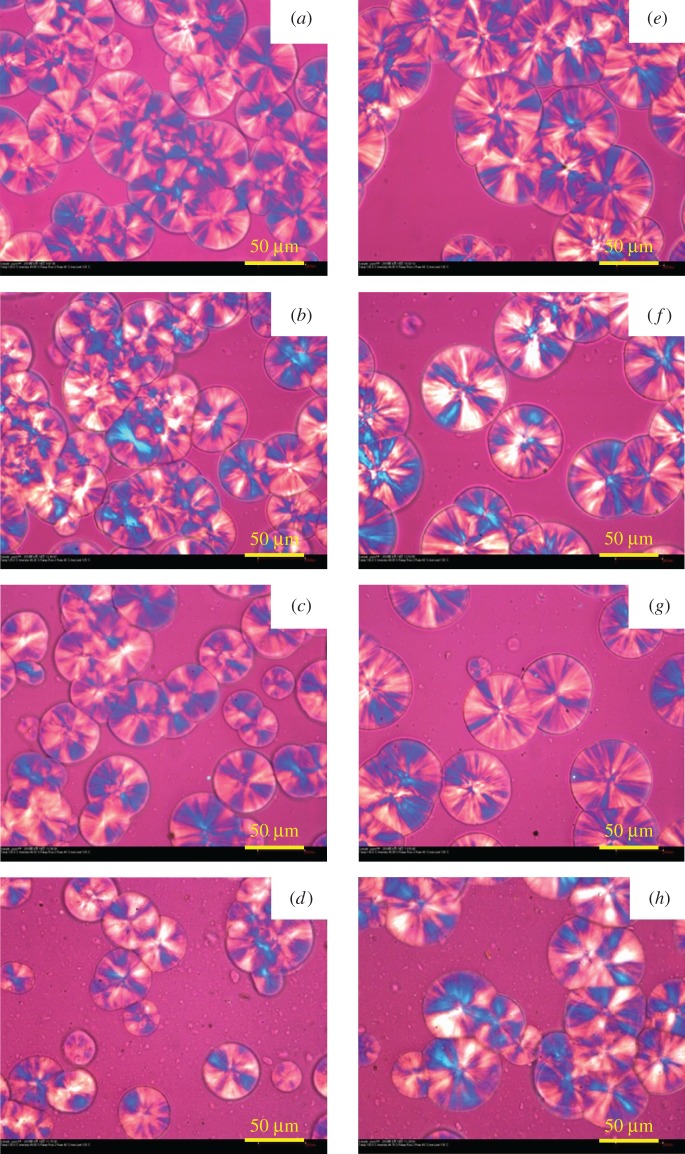
Polarized optical microscopic photographs of pure PP and nanocomposites at 125°C for 60 s: (*a*) PP, (*b*) PP/LS0.5, (*c*) PP/LS1 and (*d*) PP/LS3; at 130°C for 240 s: (*e*) PP, (*f*) PP/LS0.5, (*g*) PP/LS1 and (*h*) PP/LS3.

**Figure 12.**
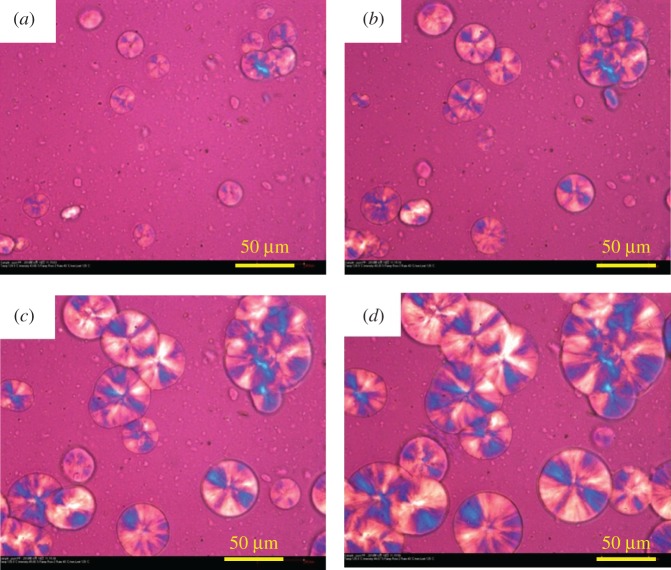
The evolution of crystalline morphology during the crystallization process of PP/LS3 at 125°C: (*a*) 25 s, (*b*) 40 s, (*c*) 60 s and (*d*) 80 s.

In order to investigate the influence of the added LDH particles on the morphology and size of spherulites of PP, POM characterization [71,72] was carried out. Figure 11 shows the POM photographs of pure PP and its nanocomposites isothermally crystallized at 125°C and 130°C, respectively. Obviously, pure PP has the typical spherulite structure. In addition, the nuclei density decreases significantly and the spherulite size slightly increases with incorporation of LDHs. But when the loading of LDHs increases to 3.0 wt%, the corresponding spherulite size appears to be smaller than that of pure PP. The reason for above phenomenon can be explained by the hindrance of nucleation in the presence of LDHs. Figure 12*a*–*d* shows the evolution of crystalline morphology during the crystallization of sample PP/LS3 at 125°C. It can be seen that the spherulites are gradually growing with increasing time.

**Figure 13.**
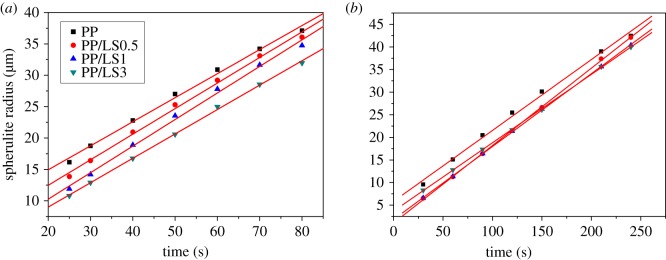
Spherulite radius versus isothermal crystallization time for pure PP and nanocomposites at (*a*) 125°C and (*b*) 130°C.

The plots of spherulite radius versus isothermal crystallization time for pure PP and PP/LDH nanocomposites are shown in figure 13. It can be seen that these plots show a good linear relationship. The spherulite growth rate *G* is obtained from the slope, as shown in table 5. It can be seen that the average spherulite growth rate decreases with increase in the isothermal crystallization temperature. In addition, at a constant temperature, the spherulite growth rate increases with the addition of LDHs, indicating that the added LDH particles might facilitate the diffusion of polymer chains into rearrangement during the crystallization process. As a result, the crystal growth rate increases. These results are in good agreement with the non-isothermal crystallization behaviour observed from DSC curves. According to previous discussion, it is known that 3.0 wt% LDHs lead to the lower spherulite size (figure 11*a*–*d*), which seems to indicate that 3.0 wt% LDHs incorporated PP nanocomposite has the lower spherulite growth rate than pure PP and this result is not consistent with that appearing in figure 13 and table 5. However, this is not the truth. In fact, high loading of LDHs have much more significant inhibitive effect on the nucleation, which correspondingly shortens the time for the spherulite to grow and hence appears to decrease the spherulite size in a short time. After extension of the growth time (240 s), the spherulite size becomes close to that of pure PP, as shown in figure 11*e*–*h*.

**Table 5.** Spherulite growth rate of pure PP and its nanocomposites at 125°C and 130°C.

sample	*G* (µm s^−1^) (125°C)	*G* (µm s^−1^) (130°C)
pure PP	0.383	0.154
PP/LS0.5	0.407	0.171
PP/LS1	0.423	0.161
PP/LS3	0.394	0.159

Above analyses show that compared with pure PP, the spherulite growth rate of PP/LDHs nanocomposites increases, while the corresponding nucleation rate decreases. However, according to DSC results, it is known that the overall crystallization rate of the PP/LDHs nanocomposite samples is higher than that of pure PP. Consequently, combining the DSC results with the POM results, it can be concluded that for PP/LDHs samples, the promotion effect of LDHs on the spherulite growth prevails against the corresponding prevention effect on nucleation, resulting in the increase in the overall crystallization rate. However, conventionally the overall crystallization rate is controlled by the heterogeneous nucleating effect and the effect of spherulite growth is very small, which could be negligible. The distinctive difference in the crystallization behaviour between our system and other conventional system may be related to the special S^3^M preparation method we used. Owing to the ingenious design and three-dimensional shear structure of mill-pan, some mechanochemical grafting reaction may take place during wet pan-milling, which could improve the compatibility between PP and LDHs and also enhance the interfacial interaction. However, during melting crystallization, the formed nano LDH layers would possibly prevent the alignment of PP macromolecular chain segments nearby due to their thermodynamic incompatibility. Therefore, at the initial nucleation stage it is difficult to form new nuclei, which need more surpercooling degree and fold surface free energy. Once the nuclei are formed, the spherulite growth rate would increase to a considerable degree because of the less steric hindrance effect between spherulites and the increase in the motion capability of PP macromolecular chains in the presence of LDHs. The difference mentioned above is also possibly related to the absence of the compatibilizer in our system, which is generally added in the other conventional systems reported in the literature.

## 4. Conclusion

The crystallization behaviour and non-isothermal crystallization kinetics of pure PP and PP/LDHs nanocomposites were investigated by using the Ozawa method, modified Avrami method as well as combined Avrami–Ozawa method. The latter two methods were successfully used to describe the non-isothermal crystallization behaviour of PP and its nanocomposites. The incorporation of nano LDH particles increases the overall non-isothermal crystallization rate. However, this increase is not caused by the heterogeneous nucleation effect of the incorporated LDHs but their promotion effect on the spherulite growth due to the lower non-isothermal crystallization temperature (*T*_p_) and the lower nucleation activity in the presence of LDHs. LDH particles are found to hinder the nucleation of PP macromolecular chains and delay the crystallization process. The effective activation energy Δ*E* for non-isothermal crystallization was evaluated by the Friedman isoconversional method. The results show that Δ*E* varies with the degree of conversion and the calculated Δ*E* of the nanocomposites is lower than that of pure PP, indicating that the nanocomposites have a higher crystallization rate. The fold surface free energy *σ*_e_ based on Hoffman–Lauritzen theory proposed by Vyazovkin was also obtained. The higher *σ*_e_ value also proves the prevention of nucleation in the presence of LDHs, which is consistent with the kinetics data. Optical microscopy observations reveal that relative to pure PP, the nanocomposite shows the enhanced crystal growth rate but the decreased nuclei density. This indicates that the presence of LDHs promotes the transportation of PP macromolecular chains from melt to crystal growth surface, which is consistent with the DSC results. In conclusion, under the effect of the pan-milling, the addition of a small amount of LDH particles shows the inhibitive effect on the nucleation and, however, would be beneficial to the crystal growth, where the effect of the latter is more remarkable, leading to an increase in the overall crystallization rate of PP/LDHs nanocomposites.

## Data accessibility

All data in this study are available in the paper.
